# Relationship between concavity of the flow-volume loop and small airway measures in smokers with normal spirometry

**DOI:** 10.1186/s12890-022-01998-w

**Published:** 2022-05-28

**Authors:** Hooria Alowiwi, Stella Watson, Kanika Jetmalani, Cindy Thamrin, David P. Johns, E. Haydn Walters, Gregory G. King

**Affiliations:** 1 Airway Physiology and Imaging Group, Woolcock Institute of Medical Research, 431 Glebe Point Rd, Sydney, NSW 2037 Australia; 2 University of Sydney, Sydney, NSW Australia; 3 Melbourne School of Population and Global Health, University of Melbourne, Parkville, Australia; 4 Menzies Medical Research Institute and School of Medicine, University of Tasmania, Hobart, TAS Australia; 5 Department of Respiratory Medicine, Royal North Shore Hospital, St Leonards, NSW Australia

**Keywords:** Small airways, Smokers, COPD, Early disease, Airflow obstruction, Physiology

## Abstract

**Background:**

There is increasing evidence of small airway abnormalities in smokers despite normal spirometry. The concavity in the descending limb of the maximum expiratory flow curve (MEFV) is a recognised feature of obstruction and can provide information beyond FEV_1_, and potentially early smoking-related damage. We aimed to evaluate concavity measures compared to known small airway measurements.

**Methods:**

Eighty smokers with normal spirometry had small airway function assessed: multiple breath nitrogen washout (MBNW) from which ventilation heterogeneity in the diffusion-dependent acinar (Sacin) and convection-dependent conductive (Scond) airways were assessed, and impulse oscillometry system (IOS) from which respiratory resistance and reactance at 5 Hz (R5 and X5) were measured. Concavity measures were calculated from the MEFV, partitioned into global and peripheral concavity.

**Results:**

We found abnormal peripheral and global concavity as well as acinar ventilation heterogeneity are common in “normal” smokers. Concavity measures were not related to either MBNW or IOS measurements.

**Conclusion:**

Abnormalities in concavity indices and MBNW or oscillometry parameters are common in smokers despite normal spirometry. However, these measures likely reflect different mechanisms of peripheral airway dysfunction.

## Background

**Fig. 1:**
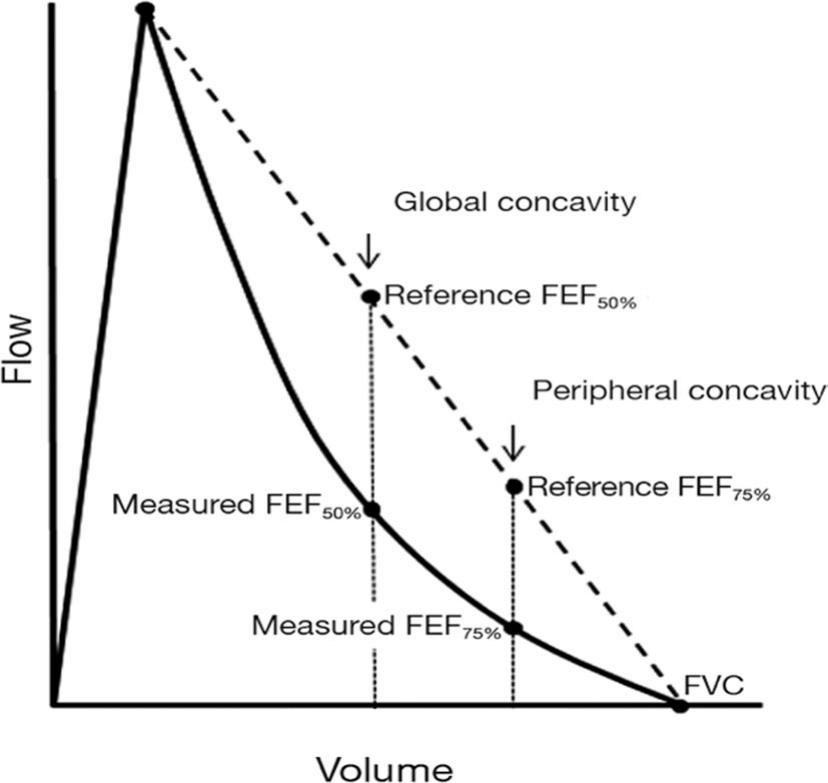
Global and peripheral concavity indices are calculated from the maximum expiratory flow-volume curve (MEFV). Global concavity is based on forced expiratory flow at 50%, and peripheral concavity is based on forced expiratory flow at 75%, of the expired FVC. The degree of concavity is compared to the “normal” reference flows at EFE50% and EFE75%

Spirometry is the “gold standard” measure of airflow obstruction, which is defined as a FEV1/FVC less than the lower limit of normal [1]. In early chronic obstructive pulmonary disease (COPD), terminal bronchiole obliteration is the initial abnormality, but will not affect the FEV1/FVC until around 2/3 of these small airways are lost since the small airways (less than 2 mm in diameter) account for little of the total airflow resistance [2]. Johns and colleagues, described two indices for estimating concavity of the expiratory limb of the flow volume curve as potential clinically useful measures of small airways disease. They defined a global index as the forced expiratory flows at 50% of the FVC (FEF50), and a peripheral index based on the FEF75, both expressed as ratios of the theoretical flows at 50% and 75% of FVC respectively, (Fig. 1) interpolated from a straight line between peak flow and RV [3]. Since there are currently complex measures of lung function that are putatively sensitive to small airway function, in particular oscillometry and multiple breath nitrogen washout (MBNW), it would be useful to know how these may relate to these global and peripheral spirometric indices.

MBNW is a complex measures of ventilation heterogeneity, where Scond is an index of this in peripheral conducting (convection dependent) airways and Sacin is an index related to the even more peripheral acinar (convection–diffusion dependent) airways. Oscillometry is another complex measure, of the respiratory system resistance (Rrs) and reactance (Xrs), reflecting especially airway caliber and oscillatory stiffness of the respiratory system, respectively. Both Rrs and Xrs, but in particular Xrs are sensitive to small airway dysfunction [4]. Results from a recent study show that 75% of smokers with normal spirometry have an abnormality in at least one MBNW or oscillometry index [5]. Our aim was to examine relationships between concavity measures and both MBNW and oscillometry parameters in current or ex-smokers with normal.

FEV1/FVC ratio from the aforementioned study [5]. We also examined concordance in abnormal results between these tests. We hypothesized that the global concavity index related to Scond and Rrs, while the peripheral index related to Sacin and Xrs.

## Methods

We analyzed previously published data of eighty current or ex-smokers (≤ 50 years of age) who had > 5 pack/year smoking history but normal spirometry. At enrollment, inclusion criteria were no history of past/present respiratory diseases or use of respiratory medications and having normal spirometry defined by post bronchodilator FEV1/FVC ratio > LLN and FEV1 > LLN. Respiratory symptoms including cough, shortness of breath, and wheeze were documented via questionnaire. Each subject was tested in a single session at the Woolcock Institute of Medical Research or Royal North Shore Hospital, Sydney, Australia. Subjects performed spirometry according to the ATS/ERS guidelines from which global and peripheral concavity indices were calculated from the maximum expiratory flow-volume curve (MEFV), as described above [3]. The upper limit of normal for post bronchodilator central concavity index is 34.8% in males and 26.3% in females; the upper limits of normal for peripheral concavity index is 61.2% in males and 63.1% in females [3]. Respiratory system impedance measures were made using a Jaeger Masterscope CT IOS (CareFusion, Hoechberg, Germany), from which Rrs and Xrs at 5 Hz (R5 and X5) were calculated. The MBNW test was preformed using an in-house-built device as previously described [5]. We used published upper and lower limits of normal to define abnormality of MBNW [6], oscillometry [7] and spirometry [8] indices. Relationships between parameters were examined by Spearman correlation coefficients. Concordance in abnormal function was evaluated using Cohen’s kappa.

## Results

**Fig. 2:**
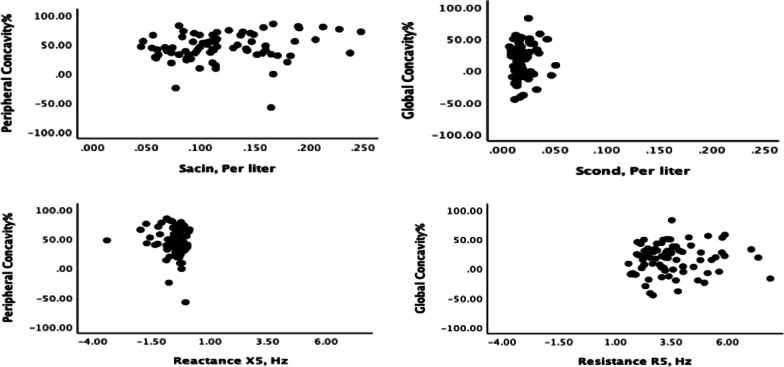
No significant correlation between peripheral concavity and Sacin (r = 0.20, *p* = 0.07), global concavity and Scond (r = 0.10, *p* = 0.34), peripheral concavity and reactance (X5) (r = − 0.09, *p* = 0.38), global concavity and resistance (R5) (r = 0.10, *p* = 0.35)

The demographics data for males (n = 51) and females (n = 29) are summarized in Table 1. Overall, abnormal peripheral concavity was found in 19/80 (23.8%) smokers and abnormal global concavity in 22/80 (27.5%) smokers. Abnormal Sacin was found in 33 (41.3%) smokers and abnormal Scond in 19 (23.8%) smokers. Only 1 and 5 participants had abnormal R5 and X5, respectively. Global concavity was unrelated to either MBNW (r_s_ = 0.10, *p* = 0.34 and r_s_ = 0.08, *p* = 0.47 for Scond and Sacin, respectively) or impedance parameters (r_s_ = 0.10, *p* = 0.35 and r_s_ = 0.02, *p* = 0.84 for Rrs and Xrs, respectively) ( Table 2, Fig. 2). Peripheral concavity was also unrelated to MBNW (r_s_ = 0.09, *p* = 0.39 and r_s_ = 0.20, *p* = 0.07 for Scond and Sacin, respectively) Peripheral concavity was unrelated to Xrs (r_s_ = − 0.09, *p* = 0.38), but weakly correlated with Rrs (r_s_ = 0.27, *p* = 0.01) (Table 2, Fig. 2). Global concavity was related to age (r_s_ = 0.27, *p* = 0.01). Peripheral concavity was related to both height (r_s_ = − 0.26, *p* = 0.02) and age (r_s_ = 0.58, *p* < 0.001). Global concavity was unrelated to smoking history (r_s_ = 0.04, *p* = 0.67), while peripheral concavity was related (r_s_ = 0.38, *p* = 0.001). There was poor concordance between concavity indices and either MBNW parameters or IOS parameters, with kappa values ranging between − 0.09 and 0.25 ( Table 3).

**Table 1:** Baseline characteristics, spirometry of smokers (n = 80)

Sex (M/F)	51/29
Age (years)	43 (11)
Height (cm)	175 (10)
BMI (kg/m.^2)^	25.2 (4.4)
Smoking (pack-years)	17.7 (10.3)
Past/current smokers	21/59
FEV_1_ (% predicted)	98 (10)
FEV_1_ (Z-score)	− 0.13 (0.81)
FVC (% predicted)	105 (12)
FVC (Z-score)	0.37 (0.90)
FEV_1_/FVC Ratio	76 (4)
FEV_1_/FVC (Z-score)	− 0.80 (0.90)
FEF_25-75_ (% predicted)	90 (23)
FEF_25-75_ (Z-score)	− 0.40 (0.81)
Global concavity (%)	17 (25)
Peripheral concavity (%)	45 (24)

There is one missing value for peripheral

Data are presented as mean (SD)

BMI, body mass index, FEF25–75, forced expiratory flow between 25 and 75% of FVC; FEV1, forced expiratory volume in

1 s; FVC, forced vital capacity

**Table 2:** Univariate correlations (Spearman correlation coefficients) between concavity indices, small airway measures, spirometry, age, and smoking history (n = 80)

Variables	Global concavity%		Peripheral concavity%	
	r_s_	*P* value	r_s_	*P* value
Scond	0.10	0.34	0.09	0.39
Sacin	0.08	0.47	0.20	0.07
R5	0.10	0.35	0.27	0.01
X5	0.02	0.84	− 0.09	0.38
FEV_1_/FVC	− 0.64	< 0.001	− 0.54	< 0.001
Age	0.27	0.01	0.58	< 0.0001
Pack/Year	0.04	0.67	0.38	0.001

There is one missing value for peripheral

*R5* resistance, *X5* reactance

**Table 3:** Abnormality overlap and concordance using Cohen’s Kappa between concavity indices and small airway measures in smokers (n = 80)

Variables	Global concavity%			Peripheral concavity%		
	Kappa	*P* value	Abnormality overlap	Kappa	*P* value	Abnormality overlap
Scond	0.24	0.02	9	0.25	0.02	8
Sacin	0.05	0.63	10	0.18	0.07	11
R5	0.06	0.10	1	0.07	0.07	1
X5	0.05	0.51	2	− 0.09	0.24	0

There is one missing value for peripheral

## Discussion

We found in this cohort of younger smokers with normal FEV1/FVC ratio, that both global and peripheral concavity indices picked up abnormalities in about a quarter of individuals, which is a similar finding to a previous unrelated evaluation[3]. However, concavity of the expiratory limb of the maximal flow-volume was unrelated to MBNW and impedance parameters and consequently there was also poor concordance between these tests in detecting abnormal airway function. Paradoxically, abnormalities in both Sacin and Scond were as prevalent as in global and peripheral indices indicating that they were all sensitive parameters, but the abnormalities did not occur in the same individuals, thus the poor concordance, suggesting that they are picking up different and quite subtle pathophysiological abnormalities. Impedance on the other hand was much less frequently abnormal. Interestingly, peripheral concavity related to smoking history, while global did not.

Ventilation in obstructive airways diseases is characterized by nonuniform lung emptying which results in reduced mid-expiratory flows in spirometry. This is manifest visually by development of concavity of the descending limb of the flow-volume curve [9]. Reduced mid-expiratory flows correlate with greater small airways disease, measured histologically in smokers [10] but is also a normal effect of ageing. The increase in lung compliance(11) with ageing explains at least part of the relationships that both concavity indices have with age. In addition, the peripheral, but not global index, correlated with smoking history. However, it is difficult to determine whether this is due partly or wholly to ageing, since age was, not surprisingly, related to pack/year history (r_s_ = 0.50, *p* < 0.001). Further, in multiple regression analysis including age and height, smoking history was no longer a significant predictor of peripheral concavity which suggests perhaps the need to develop age- and height-related prediction equations for these parameters.

There were differences between current and ex-smokers in our dataset, of the 80 smokers, 21 were former smokers. The Scond, Sacin, IOS parameters, and concavity indices were lower (i.e. less deranged) compared to that in the smoker group. Given that current smokers evidence of more peripheral airway dysfunction than ex-smokers, we suggest that abnormalities in these indices may be related ongoing smoking. The only MBNW or IOS parameter that correlated with smoking history was Sacin (r = 0.23, P = 0.03). These finding are in agreement with the previous work of Verbanck et al. In Verbanck study, she found that abnormities in Scond and Sacin occur in the absences of reduction in FEV1/FVC ratio and that with increasing pack-years of smoking, increased Scond and Sacin indicate early small airway changes [12].

The clinical usefulness of mid-expiratory flows in obstructive airways disease is uncertain. There is high concordance between FEF25-75 and FEV1/FVC ratio in detecting airflow obstruction but only 2.9% having normal FEF25-75 when the FEV1/FVC is reduced [13]. However, FEF75 was normal in 12.3% despite a reduced FEV1/FVC [13] though this may be due to abnormality in the more proximal airways and not really true COPD [3]. A strong correlation between global and peripheral indices and FEV1/FVC was evident in our study (r_s_ = − 0.64, p < 0.001 and r_s_ = − 0.54, *p* =  < 0.001, respectively) (Table 2), which suggests that they are looking at the same phenomenon but concavity is more sensitive. Mid-expiratory flows have not yet been shown to be predictive of COPD. Part of the problem may be due to its high within- and between-individual variability, and its high prevalence of abnormality in smokers [14, 15] most of whom do not develop COPD as currently defined by FEV/FVC. In contrast, imaging measures of small airways disease, lung clearance index, diffusing capacity for carbon monoxide, airway wall thickening and emphysema predict greater loss of FEV1 [16, 17].

The lack of relationship between concavity indices and MBNW or oscillometry parameters could be because spirometry is a forced manoeuvre, while MBNW and oscillometry are tidal breathing tests. If smoking altered parenchymal interdependence and airway compliance, this might translate to abnormality of forced manoeuvres but not necessarily in oscillometry. The similar prevalence of abnormality in concavity indices and MBNW parameters, but with very little overlap, is difficult to explain but suggests they represent differing functional abnormalities. The prognostic implications in terms of FEV1 decline of concavity indices, oscillometry and MNBW parameters are yet to be established but are part of ongoing longitudinal cohort research.

Although we found a high prevalence of abnormal concavity indices in this cohort, this was likely due to subject selection i.e. smokers some of whom had symptoms. Secondly, the upper limits for the concavity indices were derived healthy subjects of mean age 59 (range 40–87) years, which is significantly older than our study cohort. There may have also been differences due to geographical location (Tasmania versus Sydney). Arguably, if upper limit of normal values had been determined from a younger cohort, closer in age to this study cohort, the prevalence of abnormality may have been higher. Finally, although we found associations between concavity indices and age, height and smoking history, they were weak in nature and several correlations were explored. However, the lack of associations between concavity indices, oscillometry and MBNW parameters may also be due to the relatively small sample size given the inherent variability between tests. Given the relatively small size of this cohort, it is not possible to generalize these results and associations require further exploration.

## Conclusion

Abnormalities in both concavity indices and MBNW parameters are common in smokers with normal spirometry, but infrequent using oscillometry. However, it is somewhat disappointing that there is poor concordance between these different tests in detecting abnormal function in this group of subjects, suggesting that they likely represent different aspect of lung and airway dysfunction.

## Data Availability

The data sets generated during and/or analysed during the current study are available from the corresponding author on reasonable request.

## Abbreviations

COPD: Chronic obstructive pulmonary disease
FEV_1_: Forced expiratory volume in 1 s
FEF_50%_: Forced expiratory flow at 50%
FEF_75%_: Forced expiratory flow at 75%
FVC: Forced volume capacity
IOS: Impulse oscillometry system
MBNW: Multiple breath nitrogen washout
MEFV: Maximum expiratory flow curve
R5: Resistance at 5 Hz
X5: Reactance at 5 Hz
RV: Residual volume

## References

[1] Vogelmeier CF, Criner GJ, Martinez FJ, Anzueto A, Barnes PJ, Bourbeau J (2017). Global strategy for the diagnosis, management and prevention of chronic obstructive lung disease 2017 report: GOLD executive summary. Respirology (Carlton, Vic).

[2] Hogg JC, Macklem PT, Thurlbeck WM (1968). Site and nature of airway obstruction in chronic obstructive lung disease. N Engl J Med.

[3] Johns DP, Das A, Toelle BG, Abramson MJ, Marks GB, Wood-Baker R (2017). Improved spirometric detection of small airway narrowing: concavity in the expiratory flow-volume curve in people aged over 40 years. Int J Chron Obstruct Pulmon Dis.

[4] Bhatawadekar SA, Leary D, de Lange V, Peters U, Fulton S, Hernandez P (2019). Reactance and elastance as measures of small airways response to bronchodilator in asthma. J Appl Physiol (1985).

[5] Jetmalani K, Thamrin C, Farah CS, Bertolin A, Chapman DG, Berend N (2018). Peripheral airway dysfunction and relationship with symptoms in smokers with preserved spirometry. Respirology.

[6] Jetmalani K, Chapman DG, Thamrin C, Farah CS, Berend N, Salome CM (2016). Bronchodilator responsiveness of peripheral airways in smokers with normal spirometry. Respirology.

[7] Oostveen E, Boda K, Van Der Grinten CPM, James AL, Young S, Nieland H (2013). Respiratory impedance in healthy subjects: baseline values and bronchodilator response. Eur Respir J.

[8] Quanjer P, Stanojevic S, Cole T, Baur X, Hall GL, Culver B (2012). Multi-ethnic reference values for spirometry for the 3–95-yr age range: the global lung function 2012 equations. Eur Respir J.

[9] Miller MR, Hankinson J, Brusasco V, Burgos F, Casaburi R, Coates A (2005). Standardisation of spirometry. Eur Respir J.

[10] Berend N, Woolcock AJ, Marlin GE (1979). Correlation between the function and structure of the lung in smokers. Am Rev Respir Dis.

[11] Mittman C, Edelman NH, Norris AH, Shock NW (1965). Relationship between chest wall and pulmonary compliance and age. J Appl Physiol.

[12] Verbanck S, Schuermans D, Meysman M, Paiva M, Vincken W (2004). Noninvasive assessment of airway alterations in smokers: the small airways revisited. Am J Respir Crit Care Med.

[13] Quanjer PH, Weiner DJ, Pretto JJ, Brazzale DJ, Boros PW (2014). Measurement of FEF25-75% and FEF75% does not contribute to clinical decision making. Eur Respir J.

[14] Kwon DS, Choi YJ, Kim TH, Byun MK, Cho JH, Kim HJ (2020). FEF25–75% values in patients with normal lung function can predict the development of chronic obstructive pulmonary disease. Int J Chron Obstruct Pulmon Dis.

[15] Wafy S, Agamy G, Ali A (2016). Early spirometric changes in asymptomatic smokers; is it a time dependent?. Eur Respir J.

[16] Hoesein FAAM, Jong PAD, Lammers JWJ, Mali WP, Schmidt M, Koning HJD (2015). Airway wall thickness associated with forced expiratory volume in 1 second decline and development of airflow limitation. Eur Respir J.

[17] Zaigham S, Wollmer P, Engström G (2017). The Association of lung clearance index with COPD and FEV(1) Reduction in 'Men Born in 1914'. COPD.

